# The value of deep learning-based computer aided diagnostic system in improving diagnostic performance of rib fractures in acute blunt trauma

**DOI:** 10.1186/s12880-023-01012-7

**Published:** 2023-04-13

**Authors:** Hui Tan, Hui Xu, Nan Yu, Yong Yu, Haifeng Duan, Qiuju Fan, Tian Zhanyu

**Affiliations:** 1 Department of Radiology, Affiliated Hospital of Shaanxi University of Chinese Medicine, Xianyang, China; 2 Peter Boris Centre for Addiction Research, McMaster University & St. Joseph’s Health Care Hamilton, 100 West 5th Street, Hamilton, ON L8P 3R2 Canada; 3 Institute of Medical Technology, Shaanxi University of Chinese Medicine, Xianyang, China

**Keywords:** Rib fracture, Deep learning, Computer aided diagnostic, Diagnostic performance, Reading time

## Abstract

**Background:**

To evaluate the value of a deep learning-based computer-aided diagnostic system (DL-CAD) in improving the diagnostic performance of acute rib fractures in patients with chest trauma.

**Materials and methods:**

CT images of 214 patients with acute blunt chest trauma were retrospectively analyzed by two interns and two attending radiologists independently firstly and then with the assistance of a DL-CAD one month later, in a blinded and randomized manner. The consensusdiagnosis of fib fracture by another two senior thoracic radiologists was regarded as reference standard. The rib fracture diagnostic sensitivity, specificity, positive predictive value, diagnostic confidence and mean reading time with and without DL-CAD were calculated and compared.

**Results:**

There were 680 rib fracture lesions confirmed as reference standard among all patients. The diagnostic sensitivity and positive predictive value of interns weresignificantly improved from (68.82%, 84.50%) to (91.76%, 93.17%) with the assistance of DL-CAD, respectively. Diagnostic sensitivity and positive predictive value of attendings aided by DL-CAD (94.56%, 95.67%) or not aided (86.47%, 93.83%), respectively. In addition, when radiologists were assisted by DL-CAD, the mean reading time was significantly reduced, and diagnostic confidence was significantly enhanced.

**Conclusions:**

DL-CAD improves the diagnostic performance of acute rib fracture in chest trauma patients, which increases the diagnostic confidence, sensitivity, and positive predictive value for radiologists. DL-CAD can advance the diagnostic consistency of radiologists with different experiences.

## Introduction

Acute rib fracture (ARF) is the most common traumatic fracture in patients with the blunt chest trauma in clinical work, and accounting for about 40–80% of them [1, 2]. The mortality increased with the increase of the number of rib fractures, and thoracic trauma accounted for 25% of all trauma death [3]. Timely and accurate diagnosis of ARF is not only of great value for clinical treatment but also an important indicator for forensic disability classification which can reduce unnecessary medical disputes [4–8]. Therefore, the accurate assessment of the location and type of rib fracture is essential in an emergency. The X-ray has poor diagnostic efficiency for rib fracture, due to its low contrast resolution and overlapping structures, and the occulted and/or non-displaced fractures are usually easily missed diagnoses [9]. Multi-detector spiral CT (MDCT) is the most sensitive imaging modality for the diagnosis of ARF at present [10, 11]. Generally speaking, the 4th–8th ribs fracture are the most common; the 1st–3rd ribs are rarely fractured, but often accumulate adjacent vascular and/or brachial plexus injury; and 9th–12th ribs fractures often lead to liver and/or spleen injury [12]. Although MDCT is helpful for multi-planar reconstruction of images, the non-displaced rib fractures which are accounting for over 50% of missed rib fractures are also sometimes difficult to detect and it is very time-consuming to evaluate whether every rib is fractured on hundreds of thin-slice CT images.

Nowadays, the annual growth rate of medical imaging data in China is about 30%, while the annual growth rate of radiologists is only 4.1%which imposes huge burden for radiologists to process more image data [13, 14]. Under heavy workload, manual interpretation which relies on physician's experience is error-prone and often leads to a high rate of misdiagnosis and missed diagnosis [15, 16]. Banaste et al. reported that the missed injury rate was 530 of 5979 (8.8%) at first reading of whole-body CT in patients with multiple traumas [17]. Therefore, it is difficult for radiologists to locate rib fractures accurately and quickly, especially for interns who are inexperienced. However, the missed diagnosis of rib fracture may have important consequences for patients, clinicians and radiologists. Timely and accurate diagnosis of ARF is not only of great value for clinical treatment but also an important indicator for forensic disability classification which can reduce unnecessary medical disputes [8, 18, 19].

Deep learning (DL) is a type of artificial intelligence technique mostly used for image recognition and classification. Recently, deep learning-based computer aided diagnostic (DL-CAD) system in medical imaging have achieved the satisfactory results for image recognition which may be an effective method to resolve the current shortage of radiologists, improve the diagnostic confidence and improve productivity [20]. However, the clinical application of DL-CAD traditionally focused on the pulmonary nodules detection and measurement, hepatocellular carcinoma diagnosis, breast cancer diagnosis, and the distinction between benign and malignant tumors [21–25]. Meng et al. have developed a convolutional neural network for the classification of the type of rib fracture [26]. However, they did not evaluate the diagnostic performance of radiologists with different levels of experience with and without DL-CAD assistance. The clinical application value of DL-CAD in the detection of ARF needs to be validated. Theoretically, DL-CAD can shorten the diagnostic time, but few studies have compared the difference of reading time with and without CAD assistance.

Therefore, the purposes of this study were to validate a DL-CAD system for the detection of acute rib fractures on CT images in patients with blunt thoracic trauma and to investigate the effect of DL-CAD on interns and attending radiologists' diagnostic accuracy associated with the degree of fracture displacement by receiver operating characteristic (ROC) analysis. Our hypothesis was that the DL-CAD system specialized for the detection of ARF on CT images can improve the radiologists’ diagnostic confidence and quality, and reduce the time-consumption meanwhile regardless the complete and occult rib fracture and radiologists’ experience.

## Materials and methods

### Participants

A total of 214 cases of ARFs diagnosed by chest CT examination due to acute blunt chest trauma were collected retrospectively from July 2018 to February 2019 in our hospital, including 123 males and 91 females, age range from 21 to 89 years, with an average of (54 ± 14) years old. Acute rib fractures were defined as fractures less than 7 days after the thoracic trauma [2]. The inclusion criteria were as follows: (1) CT images were obtained within 1 week after the thoracic trauma; (2) CT imaging features of ARF, including its sharp margin, lack of periosteal reaction or callus formation [2]; (3) CT images of acute rib fracture diagnosed by two senior radiologists (with more than 15 years of experience in chest CT diagnosis). Exclusion criteria: (1) CT images of having significant motion artifacts due to unconsciousness or lack of self-control and metal internal fixation artifact that could affect the diagnosis [27]; (2) CT images of bilateral 1st–12th ribs were incompletely displayed; (3) presence of old fracture, bone destruction or bone tumor, and congenital dysplasia or rib deformity; (4) those who were lost to follow-up or refused to join the study. The Affiliated Hospital of Shaanxi University of Chinese Medicine institutional review board reviewed and approved the protocol and provided continuing oversight. All participants provided informed consent through telephone communication.

### CT examination

All cases were scanned with a 64-row spiral CT (Discovery HD 750, GE Healthcare, Wisconsin, USA). Scan position: supine position with upper arm lifted (some patients (n = 28) were naturally placed on both sides of the body due to injury in the shoulder or upper limb). Scanning range: from the thoracic opening to the 12th rib lower edge. Scanning parameters: tube voltage: 120 kVp, tube current: 20–500 mA automatic current, detector pitch: 0.85, tube rotation period: 0.6 s, reconstruction convolution kernel: Standard (n = 87) or Bone (n = 127), slice thickness: 1.25 mm. Recorded the volume CT dose index (CTDI_vol_) and dose length product (DLP) of every scanning.

### DL-CAD system

A commercial and easy-to-use DL-CAD system (InferRead CT Bone Research, Infervision, Beijing, China), which extracted image features via an artificial convolutional neural network to automatically detect rib fractures were used in our study. An information list containing all detected rib fractures for each patient were provided by the DL-CAD, and the location of each fracture on the CT images was labeled with a box.

### Reading experiment

The reading mode of this study was based on Meng et al. research [26]. Two intern radiologists (who have 1 year of experience) and two attending radiologists (who have more than 7 years of experience) participated in this reading experiment. The task was performed in two reading sessions at an interval of 4 weeks apart. At each session, all 214 cases were randomized into 7 groups, including 31 patients in groups 1, 3, 5, and 7, and 30 patients in groups 2, 4, and 6. All data sets were presented to readers in randomized order, and orders were different for every reader. In order to reduce the adverse impact from fatigue, which was usually caused by long-time consecutive reading work, the readers performed one group of reading experiment per day and thus they finished each session in a week. All readers interpreted all the cases on a picture archiving and communication system independently in the first session. And after a memory washout period of 4 weeks, the second session was implemented, in which the reader re-interpreted all cases with the assistance of DL-CAD in concurrent reading mode. During the reading procedure, the readers could adjust the window width/level and zoom in/out, or use maximum intensity projection or volume rendering if needed.

In both reading sessions, all readers were instructed to focus on detecting rib fracture. Considering that each rib has a possibility of fracture, 24 ribs for each patient were evaluated. And a 5-point Likert-scale was used to evaluate the diagnostic confidence of each rib: 1, definitely absent; 2, probably absent; 3, indeterminate; 4, probably present; 5, definitely present. For each patient, the fracture locations and diagnostic confidence score of each rib and the time-consumption for reading procedure were recorded. All readers had received DL-CAD system knowledge training before reading, and were blinded to result of the ground truth from the two senior radiologists.

### Ground truth

First, the rib fractures of all 214 participants' CT images were marked by two radiologists (engaged in musculoskeletal imaging diagnosis for more than 7 years). Two senior radiologists (Mr. JIA and Mr. DUAN, both with more than 15 years of experience in chest CT diagnosis) reviewed all the cases and their consensus diagnoses were referred as ground truth [26]. Additionally, in order to evaluate the diagnostic performance of complete rib fracture (CRF) and occult rib fracture (ORF), each fracture was classified as either complete or occult by the two senior radiologists. A CRF was confirmed if the fracture line run through the entire cortical bone with the cortex continuity completely interrupted. An ORF was referred to the bone density increasing, folding, warping, partial unconnected at external and/or internal bone cortex [29]. In order to improve diagnostic accuracy, the electronic medical record and follow up CT examination images would be reviewed in this gold standard session if needed.

### Statistical analysis

The diagnostic sensitivity, specificity, positive predictive value (PPV), diagnostic confidence and average time-consumption per case were calculated and compared between two reading sessions. Receiver operating characteristics (ROC) analysis was performed and area under the receiver operating characteristic curve (AUC) was calculated and compared. The paired sample *t* test was used to compare quantitative data, Chi-square test was used to compare the Constituent ratio data, and nonparametric Wilcoxon test was used to compare the ranked data. The P value of less than 0.05 was considered statistically significant. Statistical analysis was performed using SPSS® Statistics 19.0 (IBM Corporation, Armonk, NY; formerly SPSS Inc., Chicago, IL).

## Results

### Patients’ characteristics

A total of 214 patients with 680 ARFs were confirmed by the two senior radiologists as the ground truth, including 529 CRFs and 151 ORFs. The majority (57.5%) of ARF patients were male. The patients' age was 54.2 ± 14.7 years. The patients' trauma time to CT scan ranged from 0 to 7 days with a mean time of 4.3 days (standard deviation, 2.1 days). The CTDI_vol_ and DLP values were 7.04 ± 3.8 mGy and 308.64 ± 80.38 mGy cm, respectively. The distribution of the patient's characteristics and each rib fracture were shown in Table 1.

**Table 1:** summary of demographic characteristics of all patients

Characteristics	Values
Age (years)	54.2 ± 14.7
*Sex*	
Male	123 (57.5%)
Female	91 (42.5%)
BMI (kg/m^2^)	24.8 ± 4.9
*Fracture types*	
CRFs	529 (77.8%)
ORFs	151 (22.2%)
Trauma time to CT scan (days)	4.3 ± 2.1
*Dose parameters*	
CTDI_vol_ (mGy)	7.04 ± 3.8
DLP(mGy cm)	308.64 ± 80.38
*Fractured rib*	
1 (%)	15 (2.2%)
2 (%)	47 (6.9%)
3 (%)	74 (10.9%)
4 (%)	89 (13.1%)
5 (%)	93 (13.7%)
6 (%)	89 (13.1%)
7 (%)	85 (12.5%)
8 (%)	58 (8.5%)
9 (%)	47 (6.9%)
10 (%)	40 (5.9%)
11 (%)	26 (3.8%)
12 (%)	17 (2.5%)

*BMI* body mass index, *CTDI*_*vol*_ volume CT dose index, *DLP* dose length product

### Comparison of diagnostic performance

**Fig. 1:**
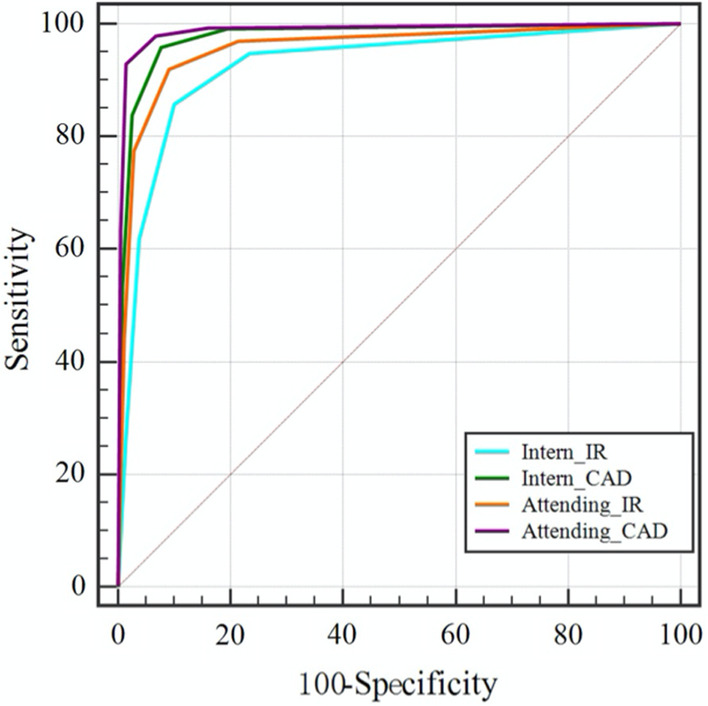
ROC curves for interns and attendings with and without the DL-CAD assistance

Totally 468 rib fractures, including 396 CRFs and 72 ORFs, were accurately detected by the interns independently. The sensitivity, specificity and PPV were 68.82%, 96.25% and 84.50%, respectively. When assisted by DL-CAD, 624 rib fractures, including 503 CRFs and 121 ORFs, were accurately detected. The sensitivity and PPV significantly increased to 91.76% and 93.17% (*P* < 0.05), respectively, while the specificity remained at similar with a value of 97.46%. Among them, the sensitivity of ORFs increased from 47.68% to 80.13% (*P* < 0.05). ROC analysis exhibited that the AUC was improved from 0.925 to 0.977 (Fig. 1). By contrast, 588 rib fractures, including 476 CRFs and 112 ORFs, were accurately detected by the attendings independently. The sensitivity, specificity and positive predictive value (PPV) were 86.47%、97.55% and 93.83%, respectively. When assisted by DL-CAD, 643 rib fractures, including 513 CRFs and 130 ORFs, were accurately detected. The sensitivity significantly increased to 94.56% (*P* < 0.05), while the specificity and PPV remained similar, with a value of98.47% and 95.67% respectively. Besides, the sensitivity of ORFs increased from 74.17% to 86.09% (*P* < 0.05). AUC was also improved from 0.955 to 0.987 (Fig. 1). Additionally, with a substantial improvement of the sensitivity and PPV for the interns, no statistically significant difference was found between the interns and attendings in all observation parameters including sensitivity, specificity and PPV when assisted by DL-CAD, which indicated that the inter-observer consistency between interns and attendings was improved (Table 2).

**Table 2:** Diagnostic performance of interns and attendings in IR and DL-CADAR

Parameters	Reading modes		*P* value
	IR	DL-CAD AR	
*Sensitivity (%)*			
Interns	68.82	91.76	0.000*
Attendings	86.47	94.56	0.045*
*Specificity (%)*			
Interns	96.25	97.46	0.621
Attendings	97.12	98.47	0.952
*PPV (%)*			
Interns	84.50	93.17	0.034*
Attendings	93.83	95.67	0.876

*IR* independent reading, *DL-CAD AR* DL-CAD assisted reading

*The difference was statistically significant (*P* < 0.05)

### Comparison of diagnostic confidence

**Fig. 2:**
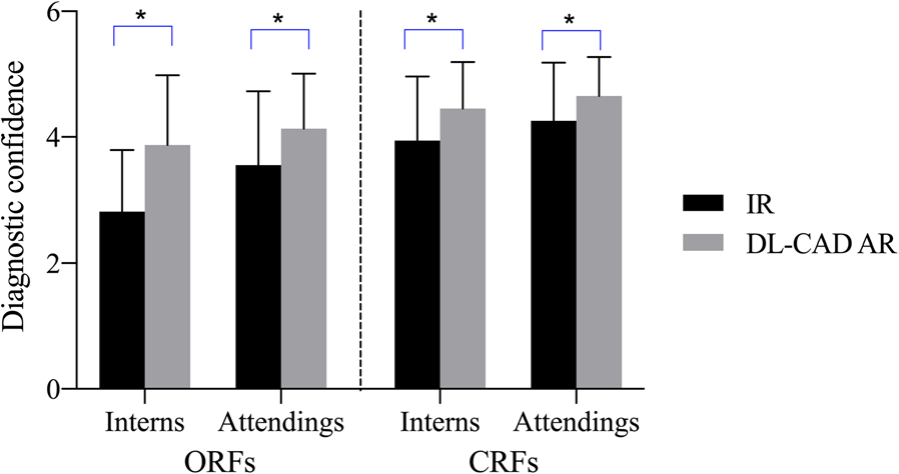
The comparison of diagnostic confidence of ORF and CRF between interns and attendings with and without the DL-CAD assistance. Note: *The difference was statistically significant (*P* < 0.05). *IR* Independent reading, *DL-CAD AR* DL-CAD assisted reading, *ORFs* occult rib fractures, *CRFs* complete rib fractures

**Fig. 3:**
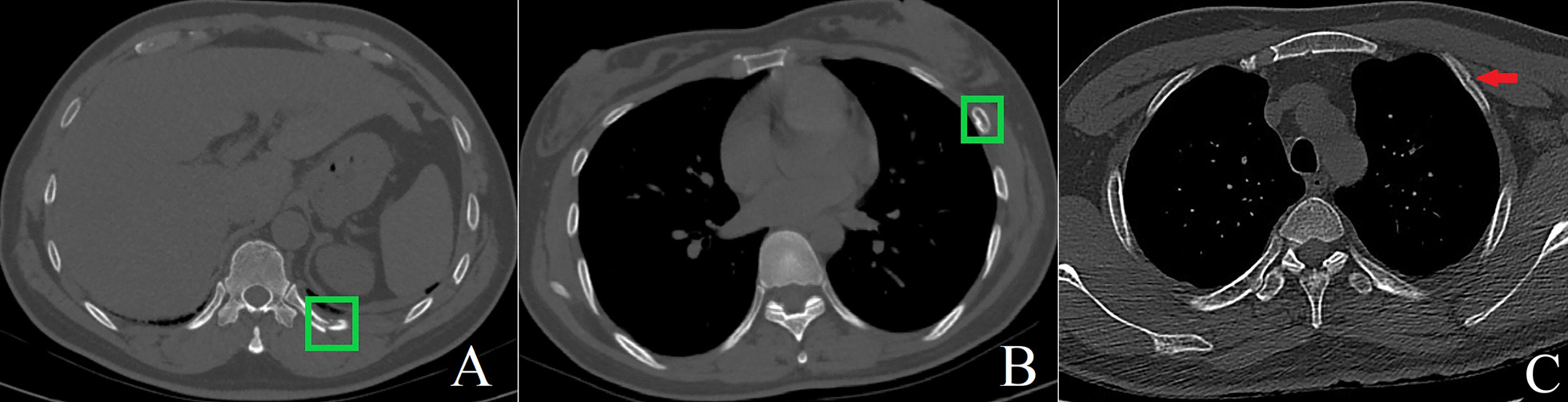
**a** A male patient (age between 40 and 45 years old) injured in a traffic accident. The interns and attending radiologists all accurately diagnosed rib fracture in two rounds of reading. The diagnostic confidence scores were all 5 points. With the assistance of DL-CAD, the reading time was shortened from 95 and 59 s to 45 s and 20 s, respectively. **b** A female patient (age between 30 and 35 years old) with blunt chest trauma. The interns and attending radiologists missed the fracture in independent reading, and the diagnostic confidence score was 1 point. With the assistance of DL-CAD, the fracture was correctly diagnosed and the diagnostic confidence increased to 4 points. **c** A 30 years old male patient with blunt on the left chest trauma, which is a false negative rib fracture image example. DL-CAD, the interns and the attending radiologists all diagnosed that the ribs were normal, but the left second external bone cortex was partially folded and warped (red arrow), and the senior radiologists diagnosed that the rib was a ORF

The average diagnostic confidence scores of the interns in the first and second session were 3.69 ± 1.12 and 4.32 ± 0.87, respectively, with a statistically significant difference (*P* < 0.05). in particularly, the average diagnostic confidence scores of CRFs and ORFs were significantly improved from 3.94 ± 1.09 and 2.27 ± 1.31 to 4.45 ± 0.79 and 3.78 ± 1.22 respectively (*P* < 0.05). The ratio of 5-points confidence (definitely present) increased from 26.47% (180/680) to 53.38% (363/680) when interns were assisted by DL-CAD. In terms of attendings, a similar tendency was found. The average diagnostic confidence scores of the first and second session was 4.11 ± 1.03 and 4.54 ± 0.72, respectively, with a statistically significant difference (*P* < 0.05). The average diagnostic confidence scores of CRFs and ORFs significantly increased from 4.26 ± 1.00 and 3.47 ± 1.15 to 4.65 ± 0.63 and 4.09 ± 1.07 respectively (*P* < 0.05). The ratio of 5-points confidence (definitely present) also significantly increased from 44.26% (301/680) to 63.53% (432/680) (Figs. 2, 3 and Table 3). Overall, compared with independent reading, both interns and attendings were significantly more confident in their diagnoses when using DL-CAD assistance.

**Table 3:** The diagnostic confidence between interns and attending radiologists with and without DL-CAD assistance

Readers	Diagnostic confidence					*P* value
	1	2	3	4	5	
*Interns*						
IR	36 (5.29%)	61 (8.97%)	163 (23.97%)	240 (35.29%)	180 (26.47%)	0.000*
DL-CAD AR	6 (0.88%)	22 (3.24%)	82 (12.06%)	207 (30.44%)	363 (53.38%)	
*Attendings*						
IR	21 (3.09%)	34 (5.00%)	98 (14.41%)	226 (33.24%)	301 (44.26%)	0.000*
DL-CAD AR	5 (0.74%)	9 (1.32%)	34 (5.00%)	200 (29.41%)	432 (63.53%)	

*IR* independent reading, *DL-CAD AR* DL-CAD assisted reading, *PPV* positive predictive value

*The difference was statistically significant (*P* < 0.05)

### Comparison of diagnostic efficiency

**Fig. 4:**
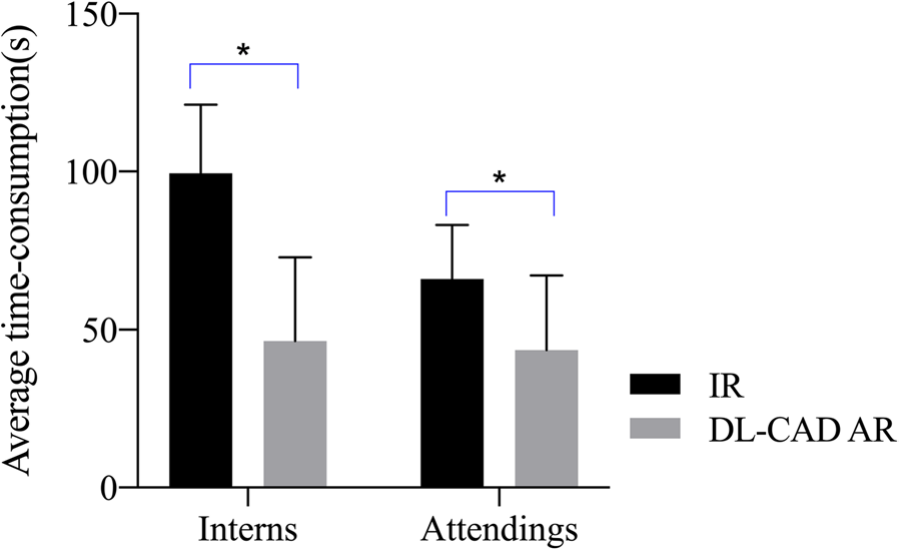
The average reading time of interns and attendings with and without the DL-CAD assistance. Note: *The difference was statistically significant (*P* < 0.05)

The average time-consumption per patient was 99.48 ± 21.69 s when interns interpreted independently in the first session, which was reduced to 46.40 ± 26.40 s with the assistance of DL-CAD in the second session, corresponding to a substantial decrease of 53.4% (*P* < 0.05). Similarly, the average time-consumption of the attendings in the first and second session was 65.96 ± 17.08 s and 43.54 ± 23.54 s respectively, which suggested a significant decrease of 34.0% was achieved when assisted by DL-CAD (*P* < 0.05) (Fig. 4).

## Discussion

In our study, with the assistance of DL-CAD, the sensitivity and PPV of interns in diagnosing ARF increased significantly from 68.82 and 84.50% to 91.76 and 93.17%, respectively (*P* < 0.05), and the sensitivity of ORFs increased from 47.68% to 80.13%. The sensitivity of attending radiologists in diagnosing ARF increased significantly from 86.47 to 94.56% (Table 2). When assisted by DL-CAD, there was no statistically significant difference in diagnostic efficiency between interns and attending radiologists. Additionally, the diagnostic confidence scores of the interns and attending radiologists in the first and second session increased significantly from 3.69 ± 1.12 and 4.11 ± 1.03 to 4.32 ± 0.87 and 4.54 ± 0.72, respectively (*P* < 0.05) (Fig. 2 and Table 3). In a word, compared with independent reading, both interns and attending radiologists were significantly more confident in their diagnoses when using DL-CAD assistance. Besides, the average time-consumption of interns and attending radiologists decreased by 53.4% and 34.0% when assisted by DL-CAD (Fig. 4).

Rib fractures are often implicated in blunt thoracic injury. A previous study suggests that patients with isolated rib fractures should be hospitalized if the number of fractured ribs is three or more. It also advocates those elderly patients with six or more fractured ribs should be treated in intensive care units due to high morbidity and mortality [30]. Another clinical study indicates that the greater number of fractured ribs correlates with higher the mortality and morbidity rates. It is vital for the emergency clinical workflow to timely and accurately identify the presence of rib injuries that require urgent attention [12].

CT examination is more effective than DR in detecting acute rib fracture. However, due to the subjective factors such as heavy workload, fatigue and in consistent diagnostic experience, diagnosis of acute rib fracture with MDCT has a high misdiagnosis rate [32]. At present, unlike fields such as lung cancer screening, where several studies about clinical application of DL-CAD have been reported [33–39], the research on the application of deep learning technology in fracture diagnosis mostly focuses on algorithms development [40]. We applied DL-CAD for the automatic detection of acute rib fractures in different reading modes to improve the diagnostic confidence, performance, and reading time of radiologists.

In order to improve diagnostic quality, all radiological reports were generated by a 2-step review system in China which was firstly written by a junior radiologist (Intern or resident) with less experience, and then confirmed by a senior radiologist (attending or more senior) with more experience. In this study, to explore the impact of DL-CAD for novel and experienced radiologists, two interns and two attending radiologists interpreted all cases independently in a blind and random way without or with the help of DL-CAD in two sessions, and the impact of DL-CAD was analyzed.

The results of our study exhibited that compared with independent diagnosis, the average diagnosis confidence of interns and attending doctors increased with the assistance of DL-CAD in a concurrent reading mode, and this was in line with recent studies. Meng et al. applied a deep learning model to detect rib fractures, and the results showed that the radiologists achieved a F1-score [26]. Generally speaking, the lack of diagnostic confidence usually leads to a more conservative assessment, and sufficient confidence helps to give clear suggestions for treatment, which could potentially reduce the excessive examination caused by follow-ups [41, 42]. It is worth noting that there are interpretation inconsistency between different diagnostic radiologists, especially for the ORFs, because of its inconspicuous image manifestations [8, 43]. In the process of clinical imaging diagnosis, it is necessary to closely combine the patient's history of trauma, including the exact location and time of trauma, or the occurrence of callus during re-examination. Besides, the diagnostic confidence of both CRF and ORF for interns and attendings were improved. Additionally, the ratio of highest diagnostic confidence (5-points) increased from 26.47% (180/680) to 53.38% (363/680) for interns, and from 44.26% (301/680) to 63.53% (432/680) for attending, respectively (*P* < 0.05). Those results indicated that the diagnostic confidence of radiologists did benefit from the assistance of DL-CAD system. This was the first report of DL-CAD that improved the diagnostic confidence of interns and attending radiologists.

In terms of diagnostic quality, the sensitivity and PPV of interns in independent diagnosis were significantly lower than those of attending radiologists. The sensitivity, specificity and PPV was improved by the assistance of LD-CAD from 68.82%, 96.25% and 84.50% to 91.76%, 93.17% and 97.46% for interns, and from 86.47%, 97.55% and 93.83% to94.56% (*P* < 0.05), 98.47% and 95.67% for attendings. Besides, much more ORFs were detected with the assistance of DL-CAD for both interns and attendings. The ROC analysis also clearly exhibited a larger AUC for both interns and attendings when using DL-CAD. Those results suggested that the diagnostic quality of radiologists with different diagnostic experience could benefit from the use of DL-CAD. To avoid false negatives, the readers could adjust the window width/level and zoom in/out, or use maximum intensity projection or volume rendering if needed during the reading procedure. In this regard, the attending radiologists who had more diagnostic experience and often did better. In addition, compared with attendings, a more substantial improvement was found for the interns. No statistically significant difference was found between the interns and attendings in all observation parameters including sensitivity, specificity and PPV when assisted by DL-CAD, which apparently resulted in a decrease of inter-observer difference. This result further evidenced that DL-CAD could significantly improve the diagnostic consistency of radiologists with different experiences. This may be because the deep learning algorithms had higher detection accuracy and could reduce the doctors' dependence on diagnostic experience. As for the diagnostic efficiency, this study showed that the average time-consumption per patient of interns and attending both decreased significantly (*P* < 0.05) when they were assisted by DL-CAD. It was probably because interpreting radiologists could locate the fracture lesion more quickly and make a diagnostic conclusion with less hesitation, with the position information of fracture provided by DL-CAD system and a higher diagnostic confidence.

Our study had some limitations. First, the major limitation of this study is its retrospective design. Most patients lack follow-up data, which might lead to underestimate the missed diagnosis rate and misdiagnosis rate. Second, in this study, the sample size was small, the CT image datasets used were only from one clinical center, and all CT images were obtained from a GE scanner. It is necessary to verify whether DL-CAD is suitable for larger sample size or CT images obtained from other vendors’ scanners. Third, the gold standard for fracture could not be obtained because few patients chose surgical treatment. The report results reviewed by two expert radiologists were used as ground truth.

## Conclusions

In conclusion, our study showed that DL-CAD system can enhance the diagnostic confidence and quality, improve the diagnostic consistency for doctors with different experiences, and reduce the time-consumption meanwhile.

## Data Availability

The data that support the findings of this study are available on request from the corresponding author.

## Abbreviations

DL-CAD: Deep learning-based computer-aided diagnostic system
ARF: Acute rib fracture
MDCT: Multi-detector spiral CT
CRF: Complete rib fracture
ORF: Occult rib fracture
PPV: Positive predictive value
ROC: Receiver operating characteristics
